# Understanding the Lived Experiences of Patients With Melanoma: Real-World Evidence Generated Through a European Social Media Listening Analysis

**DOI:** 10.2196/35930

**Published:** 2022-06-13

**Authors:** Jyoti Chauhan, Sathyaraj Aasaithambi, Iván Márquez-Rodas, Luigi Formisano, Sophie Papa, Nicolas Meyer, Andrea Forschner, Guy Faust, Mike Lau, Alexandros Sagkriotis

**Affiliations:** 1 Novartis Healthcare Pvt Ltd Hyderabad India; 2 Department of Medical Oncology Hospital General Universitario Gregorio Marañón Madrid Spain; 3 Centro de Investigación Biomédica en Red Cáncer Universidad Complutense Madrid Spain; 4 Department of Clinical Medicine and Surgery University of Naples “Federico II” Naples Italy; 5 School of Cancer and Pharmaceutical Studies King's College London London United Kingdom; 6 Department of Onco-Dermatology Toulouse Cancer Institute Toulouse France; 7 Oncology Department Toulouse University Hospital Toulouse France; 8 Department of Dermatology University Hospital Tübingen Tübingen Germany; 9 Department of Oncology University Hospitals of Leicester Leicester United Kingdom; 10 Novartis Pharma AG Basel Switzerland

**Keywords:** melanoma, social media, social media listening, real-world evidence, patient journey, cancer, mortality rate, health information

## Abstract

**Background:**

Cutaneous melanoma is an aggressive malignancy that is proposed to account for 90% of skin cancer–related mortality. Individuals with melanoma experience both physical and psychological impacts associated with their diagnosis and treatment. Health-related information is being increasingly accessed and shared by stakeholders on social media platforms.

**Objective:**

This study aimed to assess how individuals living with melanoma across 14 European countries use social media to discuss their needs and provide their perceptions of the disease.

**Methods:**

Social media sources including Twitter, forums, and blogs were searched using predefined search strings of keywords relating to melanoma. Manual and automated relevancy approaches filtered the extracted data for content that provided patient-centric insights. This contextualized data was then mined for insightful concepts around the symptoms, diagnosis, treatment, impacts, and lived experiences of melanoma.

**Results:**

A total of 182,400 posts related to melanoma were identified between November 2018 and November 2020. Following exclusion of irrelevant posts and using random sampling methodology, 864 posts were identified as relevant to the study objectives. Of the social media channels included, Twitter was the most commonly used, followed by forums and blogs. Most posts originated from the United Kingdom (n=328, 38%) and Spain (n=138, 16%). Of the relevant posts, 62% (n=536) were categorized as originating from individuals with melanoma. The most frequently discussed melanoma-related topics were treatment (436/792, 55%), diagnosis and tests (261/792, 33%), and remission (190/792, 24%). The majority of treatment discussions were about surgery (292/436, 67%), followed by immunotherapy (52/436, 12%). In total, 255 posts discussed the impacts of melanoma, which included emotional burden (n=179, 70%), physical impacts (n=61, 24%), effects on social life (n=43, 17%), and financial impacts (n=10, 4%).

**Conclusions:**

Findings from this study highlight how melanoma stakeholders discuss key concepts associated with the condition on social media, adding to the conceptual model of the patient journey. This social media listening approach is a powerful tool for exploring melanoma stakeholder perspectives, providing insights that can be used to corroborate existing data and inform future studies.

## Introduction

Melanoma is a poorly differentiated, malignant tumor arising from melanin-producing cells (melanocytes) primarily in the skin [1], with incidence increasing in the last 50 years worldwide [2]. It is an aggressive malignancy with an average 5-year survival rate of 27% once spread to distant sites [3]. According to the latest epidemiological investigations, the worldwide mortality rate of melanoma (standardized for both sexes and ages) is 0.73/100,000 [4], and it is the main cause of skin cancer–related mortality, causing up to 90% of deaths related to cutaneous malignancies [1].

Wide local excision plus sentinel lymph node dissection is the standard treatment for early-stage melanoma, while patients with regional or distant metastases present a continuing clinical challenge. With the introduction of targeted systemic therapies inhibiting kinases of the mitogen-activated protein kinase signaling pathway (specifically BRAF and MEK), as well as immune checkpoint inhibitors, long-lasting or complete remission can be achieved when treating melanoma. These treatments can stabilize the disease, reduce its burden, increase survival, and improve the quality of life (QoL) of patients with melanoma [5]. However, melanoma remains a major public health burden in Europe due to its increasing incidence, high mortality, impact on QoL, and the complexity of care for advanced stages, and it is estimated to cost >20,000 lives every year [6].

Melanoma has marked QoL implications for patients, including emotional, physical, aesthetic, and functional concerns, which are related to high levels of distress and behavioral alterations [7]. Furthermore, melanoma-related anxiety and depression have been noted among patients with high-risk primary tumors [8]. Surgery also impacts patients with melanoma both physically and emotionally [9]. These findings show that a melanoma diagnosis affects patients both physically and psychologically.

Social media provides large-scale qualitative data across countries [10]. Around 59% of European citizens use the internet to access health information, with 47%-48% using disease-specific websites (blogs and forums) and 16%-23% on social networks [11]. Social media is increasingly being used to investigate stakeholder experience in a range of health conditions, including cancer [12-15]. Social media listening (SML) may generate concepts that are more relevant to the lived experience of disease, compared with insights elucidated from interviews and focus groups [16]. After receiving a diagnosis, people often use social media platforms to share experiences and seek answers to health-related questions. Data generated on these platforms provide key stakeholder perceptions not typically shared in other real-world data (RWD) sources, clinical databases (such as registries and electronic health records), and the published literature [13]. Furthermore, insights from stakeholders other than patients (such as caregivers and family members) are also made available through SML. Therefore, SML can provide health care practitioners (HCPs) with insights into how patients and other stakeholders feel about a particular disease and the associated treatment needs [15,17,18]. It can also provide a platform for social influence, disease surveillance, risk assessment, and prevention [19].

The aim of this study was to explore how melanoma stakeholders, including patients, caregivers, and HCPs, describe their experiences on social media. Specifically, this study explored the needs and perceptions of melanoma stakeholders using SML analysis to generate insights from across European countries, in terms of treatments received, predictors of outcome, treatment effectiveness/safety, and burden of illness. The findings provide qualitative insights into the lived experience of melanoma.

## Methods

### Search Strategy

This study is a retrospective analysis of publicly available social media data, including blogs, forums, and social media platforms. Social media posts were collated between November 1, 2018, and November 30, 2020, from Austria, Belgium, France, Germany, Italy, Netherlands, Portugal, Spain, Switzerland, the Nordic countries (Denmark, Finland, Norway, Sweden), and the United Kingdom in 11 languages (Danish, Dutch, English, Finnish, French, German, Italian, Norwegian, Portuguese, Spanish, and Swedish). Search strings in each language were developed to identify conversations relevant to melanoma, using Boolean operators (AND, OR) to combine keywords (Multimedia Appendix 1).

### Data Collection

The Talkwalker Social Analytics database [20] was used to conduct searches across countries. Using the predefined search terms (Multimedia Appendix 1), social media posts were identified from in-scope geographies, and relevant posts were downloaded. The identified posts were sourced from blogs, forums, Twitter, public Facebook, and YouTube. Relevant forums and blog posts were identified using local online community websites and discussion boards (including Healthunlocked, Mumsnet, Medicitalia, 9lives, and Frauenselbsthilfe; Multimedia Appendix 2).

### Ethical Considerations

Even though social media posts are in the public domain, SML studies raise unique ethical challenges, as individuals do not formally consent to the use of their data in the research. Currently, there is little guidance on the lack of consent or anonymity of participants in social media research. However, recommendations include ensuring that the data collected answer specific research questions and presenting data in a way that avoids participant identification [21]. Appropriate steps were taken in this study to follow these recommendations. To anonymize publicly reported posts, information that could identify individual patient or caregivers (such as usernames) was removed before analysis.

### Data Analysis

The raw data set was further contextualized by excluding conversations irrelevant to the study. This was done by both an automated relevancy approach (containing keyword-based relevancy algorithms) and a manual review against predefined criteria (Multimedia Appendix 3). This relevancy check ensured conversations provided relevant insights to the patient journey stage and other patient-centric topics.

An iterative random sampling technique was employed to reduce the number of posts as per the agreed proportions of social media records by country (and their respective channels), based on the amount of data available in each country. For countries with high data volume, sampling reduced the number of relevant posts from stakeholders to ensure that a manageable amount of data were obtained for manual review. For countries with low volume, data were taken without sampling. Relevant posts were tagged by channel type and, where possible, categorized by stakeholder (patients, caregivers, family and friends, HCPs, and others, as based on the language used in the post; eg, “I have melanoma” and “I have been diagnosed with this condition”), gender (taking into account profile pictures and content using gender labels such as “daughter,” “father,” “he”/”she,” and “lady,” for example), and age group (specific mention of age in the post). A deep dive into the filtered data set was then conducted to investigate research domains listed in the inclusion criteria (Multimedia Appendix 3). The benefit of an automated methodology is that it allows large amounts of data to be analyzed quickly and efficiently to dismiss irrelevant posts. Using this approach does, however, pose the risk that some relevant posts may have been missed, as the nuances of human expression may not have been captured in some/all cases. The sentiments toward a given treatment were also judged based on the language used to describe the experiences.

## Results

### Overview of Social Media Posts

A total of 182,400 social media posts were extracted in the initial search using the predefined keyword strings (Multimedia Appendix 1), with 2547 posts identified as relevant to the study objectives (Multimedia Appendix 4). The random sampling methodology selected 864 relevant posts for further analysis (Multimedia Appendix 4).

Twitter emerged as the most commonly used social media channel (n=129,504, 71% contribution to posts), compared with blogs (n=31,008, 17%), forums (n=20,064, 11%), and other platforms (n=1,824, 1%). Most of the posts originated from the United Kingdom (n=69,321, 38%), followed by Spain (n=29,184, 16%), Italy (n=23,712, 13%), France (n=20,064, 11%), and Germany (n=20,064, 11%).

**Figure 1:**
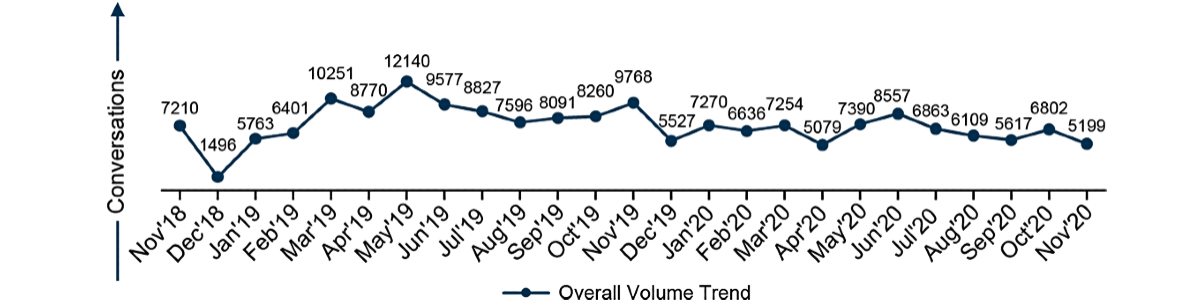
Data volume trend.

 A peak in social media discussion was observed in the spring months of May 2019 (12,140 conversations) and June 2020 (8557 conversations; Figure 1). Fewer posts originated from Nordic countries (n=7296, 4%) and the Netherlands (n=5472, 3%), while posts from Belgium, Switzerland, Portugal, and Austria, each contributed 1% (n=1824) of the total posts (Table 1). Of the 864 analyzed posts (Multimedia Appendix 4), 536 (62%) were categorized as coming from individuals who had melanoma, while 190 (22%) originated from caregivers, 104 (12%) from friends and family, 17 (2%) from HCPs, and a further 17 (2%) from other individuals.

Malignant and metastatic disease accounted for 77% (181/235) of the melanoma types mentioned (Multimedia Appendix 5). Advanced stage melanoma (which included the terms “stage IV,” “late stage,” and “metastatic stage” disease), was the most frequently discussed disease stage (154/245, 63%; Multimedia Appendix 5). Conversations were slightly more female-led (422/768, 55%), which was consistent for most countries, except in Nordic countries, where male-led conversations were more common (34/49, 69%), and Spain, where the gender split was 50% (72/145; Multimedia Appendix 5). More males in Switzerland also contributed to conversations, but the overall social media population size where gender could be determined was small (n=17). Most individuals (53%) were aged between 31 and 50 years (Multimedia Appendix 5).

**Table 1:** Country of origin of social media posts and percentage usage per social media platform.

Country			Percentage of posts, n (%)
**United Kingdom**			69,321 (38)
	Twitter	61,696 (89)	
	Blogs	4852 (7)	
	Forums	2080 (3)	
**Spain**			29,184 (16)
	Twitter	26,557 (91)	
	Blogs	2334 (8)	
	Forums	0	
**Italy**			23,712 (13)
	Twitter	11,145 (47)	
	Blogs	9959 (42)	
	Forums	2134 (9)	
**France**			20,064 (11)
	Twitter	13,644 (68)	
	Blogs	4815 (24)	
	Forums	1404 (7)	
**Germany**			20,064 (11)
	Twitter	11,236 (56)	
	Blogs	5217 (26)	
	Forums	3612 (18)	
**Nordic countries^a^**			7296 (4)
	Twitter	4961 (68)	
	Blogs	1678 (23)	
	Forums	730 (10)	
**Netherlands**			5472 (3)
	Twitter	3119 (57)	
	Blogs	1313 (24)	
	Forums	1040 (19)	
**Belgium**			1824 (1)
	Twitter	1496 (82)	
	Blogs	328 (18)	
	Forums	0	
**Switzerland**			1824 (1)
	Twitter	1532 (84)	
	Blogs	237 (13)	
	Forums	55 (3)	
**Portugal**			1824 (1)
	Twitter	1094 (60)	
	Blogs	693 (38)	
	Forums	0	
**Austria**			1824 (1)
	Twitter	967 (53)	
	Blogs	438 (24)	
	Forums	401 (22)	

^a^Denmark, Finland, Norway, Sweden.

### The Patient Journey in Melanoma

**Figure 2:**
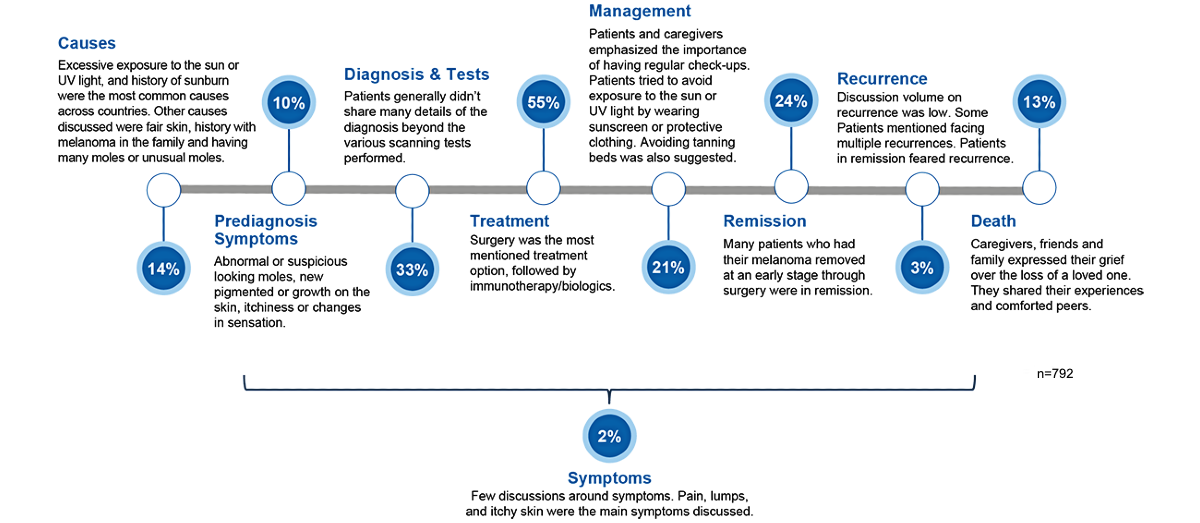
Percentage of posts for each stage in the patient journey.

This study provided key insights into the patient journeys of those living with melanoma. Treatment (436/792, 55%), diagnosis and tests (261/792, 33%), and remission (190/792, 24%) were the most frequently discussed melanoma-related topics (Figure 2). Discussions around the causation of melanoma contributed to 14% (111/792) of patient journey-related posts (Figure 2), where excessive sun or UV light exposure constituted the majority of discussions (n=95, 87%). Other causes discussed included genetics, such as having fair skin or a family history of melanoma (n=12, 11%) and having many/unusual moles (n=9, 8%).

Only 2% (16/792) of posts referred to postdiagnosis symptoms, with a further 10% (79/792) discussing prediagnosis symptoms (Figure 2). New pigmented growths on the skin (n=30, 38%), suspicious-looking moles (n=20, 25%), and darkening of the skin (n=8, 10%) were the most frequently mentioned prediagnostic symptoms (n=79). The most frequently mentioned postdiagnostic symptoms (n=14) were pain (n=5, 36%) and hardened nodules under the skin (n=3, 21%). Most discussions on diagnosis and tests were around confirmed diagnosis (92/255, 36%; Multimedia Appendix 6). Biopsy (46/255, 18%) was the most commonly mentioned confirmatory diagnostic test (Multimedia Appendix 6). Only 1% (n=10) of posts mentioned mutations (most commonly *BRAF*, *MEK*, and *CDKN2A*). A number of posts (n=170) discussed disease management and highlighted regular skin checks (n=59, 35%), avoiding the sun (n=46, 27%), and applying sunscreen (n=36, 21%). Conversations also mentioned avoiding sunbeds (n=22, 13%), which mostly originated from the United Kingdom (19/22 posts).

Multimedia Appendix 7A provides an overview of the melanoma treatments reported in the social media posts analyzed. The most frequently reported treatment was surgery (293/437, 67%), followed by immunotherapy (52/437, 12%), radiotherapy (22/437, 5%), and targeted therapy (17/437, 4%). Treatment sequence (139/295, 47%) and efficacy (133/295, 45%) were the most commonly discussed topics regarding melanoma treatment features (Multimedia Appendix 8). Treatment posts were dominated by first-line (1L) discussions (n=131), which were mostly about surgery (n=94, 72%; Multimedia Appendix 7). Few negative sentiments were associated with posts discussing surgery (n=9, 3%), which was the lowest among all treatments mentioned (Multimedia Appendix 7B). Although treatment-related discussions mentioning chemotherapy were low (n=13, 3%; Multimedia Appendix 7A), this was the treatment type with the highest associated negative sentiment (n=6, 45%; Multimedia Appendix 7B). In posts that discussed disease end points (n=226), remission/cure (referred to as “being all clear” or “finished with years of check-ups”) was the main clinical end point discussed by stakeholders (n=169, 75%), with prolonged survival (n=34, 15%) and morbidity/mortality (n=18, 8%) as the other two most frequently mentioned end points.

### Impacts of Melanoma

A total of 255 posts referred to the impacts that melanoma had on individuals’ QoL. The social media population discussed emotional (n=178, 70%), physical (n=61, 24%), social (n=43, 17%), and financial (n=11, 4%) impacts. Frequently mentioned emotional impacts in conversations (Table 2) were negative thoughts, including feeling low/upset/sad (n=59, 33%) and being affected emotionally (n=44, 25%), anxiety (n=30, 17%), distress (n=25, 14%), and fear (n=23, 13%). Melanoma stakeholders also reported being affected physically (n=21, 34%), having social behavioral changes/affected social life (n=18, 42%), and facing treatment expenses (n=6, 55%). Table 2 outlines the type of impacts of melanoma reported on social media.

**Table 2:** Impacts of melanoma (N=225) reported on social media.

Type of impact			n (%)
**Emotional impact (n=173)**			
	Feeling low/upset/sad	59 (33)	
	Affected emotionally (in general)	45 (25)	
	Anxiety	30 (17)	
	Distress	25 (14)	
	Fear	23 (13)	
	Negative feelings	12 (7)	
	Conscious about looks	7 (4)	
	Confused	5 (3)	
	Change in outlook on life	5 (3)	
	Depression	4 (2)	
**Physical impact (n=61)**			
	Affected physically (in general)	21 (34)	
	Issues due to pain	11 (18)	
	Movement issues	8 (13)	
	Feeling weak/tired/exhausted	8 (13)	
	Scar	7 (11)	
	Struggling with side effects of medications	6 (10)	
	High risk for COVID-19 infection	5 (8)	
	Cannot wear revealing clothes	3 (5)	
	No comfort	2 (3)	
	Insomnia	2 (3)	
	Cannot manage household work	1 (2)	
	Unable to do daily activities	1 (2)	
**Social impact (n=43)**			
	Social behavioral changes	18 (42)	
	Affected social life	18 (42)	
	Affected work	4 (9)	
	Affected school	2 (5)	
	Avoided by others/lost social media followers	1 (2)	
	Affected family life	1 (2)	
**Financial impact (n=11)**			
	Finding treatment expensive	6 (55)	
	Looking for financial support for treatment	4 (36)	
	Struggling with insurance coverage	1 (9)	

A lack of available or effective treatments (30/121, 25%), access to good HCPs/treatments (30/121, 25%), and safe access to care during the COVID-19 pandemic (25/121, 21%) emerged as key unmet needs of melanoma stakeholders (Multimedia Appendix 9). Concerns were expressed around the impact of COVID-19 on patients with melanoma, including changes to medical appointments, safe access to treatment, and self-isolation. In total, 5% (n=295) of posts on treatment features mentioned treatments being cancelled/postponed/rescheduled, with this being attributed to the pandemic in many countries, and 21% (n=121) of posts discussing unmet needs mentioned problems with safe access to treatment during the COVID-19 pandemic. This was a key unmet need in Belgium, France, Spain, and the United Kingdom.

## Discussion

### Principal Findings

This study identified key concepts relevant to individuals living with melanoma, providing qualitative insights into how the patient journey is discussed online by multiple stakeholders across Europe. A peak in social media discussion was observed on May 13, 2019, which was World Melanoma Day. Interestingly, a peak in posts about melanoma was observed in the early summer of both 2019 and 2020, which may coincide with the promotion of prevention resources ahead of the summer months in the northern hemisphere (such as May being Melanoma and Skin Cancer Awareness Month). European countries with larger population sizes (United Kingdom, Spain, Italy, France, and Germany) contributed to the majority of posts included in the study (89% in total), compared to countries with smaller population sizes (Austria, Belgium, Netherlands, Portugal, Switzerland and the Nordic countries, which contributed to 11% of the total posts).

Key topics highlighted in this study included melanoma treatment and diagnosis, as well as patient QoL. This complements a review of the specific communication needs of cancer patients (including melanoma) from semistructured interviews, focus groups, and questionnaire surveys, which revealed that the main discussion needs were disease-related information and psychological support [22]. Treatment sequencing, in terms of how patients were treated in 1L and later lines of therapy, was the most common treatment feature discussed. A therapy change is often initiated when a treatment fails, is not well-tolerated, or following disease relapse, suggesting that these are experiences that melanoma stakeholders are eager to discuss. Surgery was the most frequently mentioned treatment (particularly in 1L) and had the highest number of positive mentions, which was possibly attributable to its curative effects. Chemotherapy was often associated with negative sentiments, perhaps due to its side effects or noncurative nature. It potentially appears that positivity was driven by the effectiveness of the treatment, while negativity was due to patients experiencing side effects or low efficacy. Among the other treatments mentioned, immunotherapy, radiotherapy, and targeted therapy were also discussed on social media, which is not surprising given the prevalence of their use in the melanoma treatment landscape [23]. Across all treatment types, stakeholders rarely attached a sentiment while discussing specific treatment types, brands, or molecules. Tumor biopsy was the most frequently mentioned diagnostic test for melanoma. Discussions around 1L treatments and diagnosis may be indicative of patients, caregivers, and family members searching for information about melanoma online following an initial diagnosis. This highlights the important role of HCPs in providing detailed information about melanoma early in the treatment journey. Melanoma stakeholders also discussed impacts of the condition; emotional impacts were frequently mentioned, with many expressing negative thoughts.

There is currently limited qualitative research on melanoma in the social media population. Studies using patient narratives obtained from cancer support organization websites and web-based forums have highlighted the psychosocial and emotional impact following a melanoma diagnosis [24-26], consistent with the findings from this SML study. Similarly, proactive management of the condition and treatment by patients with melanoma have also been reported online [24]. Many of the topics identified by SML were consistent with those reported in other qualitative studies, in particular interviews of melanoma stakeholders [27-30]. These topics include the symptoms discussed, which, not surprisingly, are dominated by skin changes [27] and patients engaging in activities to prevent recurrence, including sun avoidance/protection [28,29]. Negative emotional impact, anxiety, distress, and fear were identified as the major impacts of living with melanoma. This is consistent with other qualitative studies, highlighting the emotional impact of the treatment journey for patients with melanoma [24,25,27,28,31,32]. Systematic reviews of qualitative and quantitative studies demonstrate that major unmet psychological needs are reflective of the emotional impact of melanoma on patients [32]. Taken together, the high level of emotional impacts identified from this SML analysis and other studies emphasizes the acute need for emotional support for patients with melanoma. This is an important finding given the consequences that negative emotional impacts, such as depression, can have on increasing cancer mortality [33]. It is also noteworthy that in addition to the psychological and emotional impacts commonly associated with melanoma and its treatment journey, this study highlights important unmet needs for patients with melanoma that might have been specifically affected by delayed cancer diagnosis and management due to the COVID-19 pandemic, a concern also shared by HCPs [30]. In fact, almost a quarter of posts, especially in Belgium, France, Spain, and the United Kingdom, were concerned with safe access to treatment during the COVID-19 pandemic. It is also probable that the pandemic might have caused heightened levels of anxiety and an overall negative emotional impact for patients with melanoma and their caregivers. The findings from this study contribute to the conceptual model of the melanoma patient journey and treatment landscape and provide knowledge on how stakeholders discuss key concepts associated with the condition. SML data provide unfiltered and uninfluenced insights [13], which can help enhance HCP-patient communication. Most SML discussions were around melanoma management and treatment rather than the early stages of disease prevention, symptom identification, and diagnosis. This might be due to the fact that a relatively large proportion (181/235, 77%) of discussions were around malignant and metastatic disease where treatment and management might be the highest priority. On the other hand, patients who were in remission or who had removed their melanomas successfully through surgery at an early disease stage were more likely to engage in discussions around melanoma awareness, for example, by promoting regular checks, banning tanning beds, and reducing sun exposure. Communication issues between patients with melanoma and their treating clinicians, particularly around informational needs at diagnosis, have been identified before in a United Kingdom–based study [34]. SML identified diagnosis as a popular discussion topic among melanoma stakeholders, suggesting that patients may have enhanced informational needs at diagnosis. Aside from helping to improve HCP-patient communication priorities, SML studies can also inform the modification of patient-reported outcome (PRO) measures to help evaluate the QoL of patients living with melanoma. This can, in turn, inform adequate measurement of QoL-related parameters in clinical trials and other research studies.

### Limitations

The social media population may not be representative of the whole community affected by melanoma. In this study, most participants were between 31 and 50 years of age, and while melanoma disproportionately affects younger people compared with other solid tumors [35], this demographic may be reflective of older people being frequently underrepresented on social media. The SML analysis comprised of a mixed population in terms of disease stage; therefore, it is challenging to identify the different needs of patients with late-stage versus early-stage melanoma due to the lack of patient-level data. Furthermore, the data set does not distinguish between treatments used in different melanoma settings (such as adjuvant or metastatic), and this may impact the interpretation of treatment discussions, including certain treatment features and treatment sequence. While there is an inherent methodological constraint of not having standardized measures to assess the severity of QoL concerns, SML provides a valuable source of information to identify relevant health-related QoL aspects, which could be cross-referenced with current QoL tools and questionnaires to potentially improve the validity of PRO measures [26].

All data were retrospectively collected from social media posts in the public domain. As a result, demographic and clinical information of the social media population could not always be obtained or confirmed. For example, it was not possible to substantiate that all individuals were posting on a confirmed melanoma diagnosis. Therefore, it must be acknowledged that some data may be incorrectly categorized. For example, identifying gender through pictures, pronouns, or family relationships is not necessarily a reliable method to infer a male or female identity. Although the accuracy of correct gender assignment has been noted to be as high as >90% in some studies, other traits including age can be more challenging to predict [36].

### Conclusions

Melanoma has a significant impact on people’s daily lives; stakeholders affected by melanoma experience significant emotional impacts that affect their QoL. In particular, 1L melanoma treatments were frequently discussed online, especially surgery, which was often associated with positive sentiments. Despite the aforementioned limitations, the findings from this study were consistent with published evidence, supporting insights captured by other RWD studies. This suggests that SML approaches can identify topics that provide person-focused, real-world insights into the lived experiences of melanoma that are not typically available in the published literature and that can be used to corroborate existing data and inform future studies. To monitor what melanoma stakeholders are most concerned about, it is advisable to repeatedly conduct online analysis such as the one in this study. At the same time, efforts should be made to increase the visibility of reliable data sources (such as links to treatment guidelines) on social media.

## Appendix

Multimedia Appendix 1 Social media search strings.

Multimedia Appendix 2 Sources of forum and blog posts.

Multimedia Appendix 3 Predefined inclusion/exclusion criteria.

Multimedia Appendix 4 Analysis process for post relevancy.

Multimedia Appendix 5 Stakeholder demographics by country.

Multimedia Appendix 6 Percentage of melanoma stakeholder discussion on diagnosis and tests.

Multimedia Appendix 7 Melanoma treatments reported in social media posts and associated sentiment. (A) Type of melanoma treatments reported. (B) Melanoma treatments as discussed by sentiment.

Multimedia Appendix 8 Melanoma treatment discussions.

Multimedia Appendix 9 Key unmet needs of melanoma stakeholders.

## Abbreviations

HCP: health care practitioner
PRO: patient-reported outcome
QoL: quality of life
RWD: real-world data
SML: social media listening
1L: first-line
