# Supplementary material for: Understanding the Lived Experiences of Patients With Melanoma: Real-World Evidence Generated Through a European Social Media Listening Analysis
Source: JMIR Cancer. 2022 Jun 13;8(2):e35930. doi: 10.2196/35930 (PMC9237767; doi:10.2196/35930)
Supplement: Multimedia Appendix 1 [file cancer_v8i2e35930_app1.docx]

*Multimedia Appendix 1. Social Media Search Strings.*

| **Language** | **Search Terms Used*** |
| --- | --- |
| **Danish** | (Melanom* OR Melanom OR #Melanom* OR "#Metastatiskmelanom" OR Metastatiskmelanom OR "#mMelanom" OR mMelanom* OR "#amelanotiskMelanom" OR amelanotiskMelanom* OR "#invasivMelanom" OR overfladiskspredningmelanom OR overfladiskspredningmelanom OR overfladisktspredendemelanom OR nodularmelanom OR lentigomalignamelanom OR malignamelanom OR ondartetmelanom OR "#ondartetmelanom" OR Akrallentiginousmelanom OR nevoidmelanom OR spitzoidmelanom OR desmoplastiskmelanom OR kutantmelanom) |
| **Dutch** | (Melanoom* OR melanomen OR #Melanoom* OR "#MetastatischMelanoom" OR "metastatischmelanoom" OR "#mMelanoom" OR mMelanoom OR "#amelanotischmelanoom" OR "amelanotischmelanoom" OR "#invasiefMelanoom" OR superficialspreadingmelanoma OR "nodulairmelanoom" OR "lentigomalignamelanoom" OR "malignemelanoom" OR "malignemelanomen" OR "kwaadaardigmelanoom" OR "#kwaadaardigmelanoom" OR "#malignemelanoom" OR "Acraallentigineusmelanoom" OR "amelanotischmelanoom" OR "nevoïdmelanoom" OR "spitzoïdmelanoom" OR "desmoplastischmelanoom" OR huidmelanoom) |
| **English** | (Melanoma* OR #Melanoma* OR "#MetastaticMelanoma" OR metastaticmelanoma OR "#mMelanoma" OR mMelanoma OR "#amelanoticMelanoma" OR amelanoticMelanoma OR "#invasiveMelanoma" OR superficialspreadingmelanoma OR nodularmelanoma OR lentigomalignamelanoma OR malignamelanoma OR malignantmelanoma OR "#malignantmelanoma" OR Acrallentiginousmelanoma OR amelanoticmelanoma OR nevoidmelanoma OR spitzoidmelanoma OR desmoplasticmelanoma OR cutaneousmelanoma) |
| **Finnish** | (Melanooma* OR #Melanooma* OR melanoomi* OR tummasolusyöpä* OR "#metastaattinenmelanooma" OR metastaattinenmelanooma OR “levinyyt* melanom*” OR “levinn* melanoom*” OR "amelanoottinen melanooma" OR “invasiivinen melanooma” OR “pinnallises* leviävä* melanoom*” OR “nodulaarin* melanoom*” OR “nodulaaris* melanoom*” OR “lentigo maligna melanooma” OR jättikesakkomelanoom* OR “akraalinen melanooma” OR “amelanoottinen melanooma” OR “nevoidi melanooma” OR “spitzoidi melanooma” OR “desmoplastinen melanooma” OR “desmoplastic* melanoom*” OR ihomelanoom*) |
| **French** | (mélanome* OR #Mélanome* OR #Mélanomemétastatique OR "mélanomemétastatique" OR "#mMélanome" OR mMélanome OR "#mélanomeamélanotique" OR "mélanomeamélanotique" OR "#mélanomeachromique" OR "mélanomeachromique" OR "#mélanomeinvasif" OR "mélanomesuperficielextensif" OR "mélanomesàextensionsuperficielle" OR "mélanomenodulaire*" OR "lentigomalignamélanome" OR "mélanomesurmélanosedeDubreuilh" OR "mélanomedelentigomalin" OR "mélanomemalin*" OR "#mélanomemalin" OR "mélanomeacrallentigineux" OR "mélanomeacrallentigineux" OR "mélanomenévoïde" OR "mélanomespitzoïde" OR "mélanomedesmoplastique" OR "mélanomecutané") |
| **German** | (Melanom* OR #Melanom* OR "#metastasiertesMelanom" OR "metastasiertesMelanom" OR "malignesMelanom" OR "malignesMelanom" OR "malignMelanome" OR (amelanotisch* AND Melanom*) OR "#AmelanotischesMelanom" OR "#invasivesMelanom" OR (invasiv* AND Melanom*) OR "lentigomalignaMelanom" OR ("lentigo maligna" AND Melanom*) OR "oberflächlichspreitendesMelanom" OR (amelanotisch* AND Melanom*) OR "akrallentiginösesMelanom" OR (Akrolentiginös* AND Melanom*) OR (noduläre AND Melanom*) OR (nävoid* AND Melanom*) OR (spitzoid* AND Melanom*) OR (desmoplastisch* AND Melanom*) OR Hautmelanom) |
| **Italian** | (Melanoma* OR #Melanoma* OR "#melanomametastatico" OR "melanoma metastatico" OR "#mMelanoma" OR mMelanoma OR "#melanomaamelanotico" OR "melanoma amelanotico" OR "#melanomainvasivo" OR "melanoma a diffusione superficiale" OR nodulo OR "melanoma nodulare" OR lentigomalignamelanoma OR malignamelanoma OR "melanoma maligno" OR "#melanomamaligno" OR Acrallentiginousmelanoma OR melanomaanemalotico OR melanomanevoide OR "melanoma nevoide" OR melanomaspitzoide OR "melanoma spitzoide" OR melanomadesmoplastico OR "melanoma desmoplastico" OR melanomacutaneo OR "melanoma cutaneo") |
| **Norwegian** | (Melanom* OR Melanom OR #Melanom* OR "#Metastatiskmelanom" OR Metastatiskmelanom OR "#mMelanom" OR mMelanom* OR "#amelanotiskMelanom" OR amelanotiskMelanom* OR "#invasivMelanom" OR Superfisieltspredendemaligntmelanom OR overfladiskspredningmelanom OR Superfisieltspredendemelanom OR nodularmelanom OR lentigomalignamelanom OR malignamelanom OR ondartetmelanom OR "#ondartetmelanom" OR Akrallentiginousmelanom OR nevoidmelanom OR spitzoidmelanom OR desmoplastiskmelanom OR kutanmelanom) |
| **Portuguese** | melanoma* OR #melanoma OR melanomasuperficial OR melanomanodular OR melanomacutaneo OR melanomamaligno OR melanomalentiginoso* OR melanomametastatico |
| **Spanish** | (Melanoma* OR #melanoma* OR #Melanomametastásico OR #Melanomametastasico OR melanomametastásico OR melanomametastasico OR metastásismelanoma OR metastasismelanoma OR melanomamelanótico OR melanomamelanotico OR amelanóticomelanoma OR amelanoticomelanoma OR "melanomainvasivo" OR "melanomaextendido" OR "melanomanodular" OR "melanomalentigomaligno" OR "melanomamaligno" OR "melanomaamelanótico" OR "melanomaamelanotico" OR "melanomanevoide" OR "melanomaspitzoide" OR "melanomadesmoplásico" OR "melanomadesmoplasico" OR "melanomacutáneo" OR "melanomacutaneo") AND sourcegeo_accuracy:high |
| **Swedish** | (Melanom* OR Melanom OR #Melanom* OR "#Metastatiskmelanom" OR Metastatiskmelanom OR "#mMelanom" OR mMelanom* OR "#amelanotiskMelanom" OR amelanotiskMelanom* OR "#invasivMelanom" OR ytligspridandemelanom OR ytligtspridandemelanom OR Ytligtväxandemelanom OR superficielltspridandemelanom OR ssmelanom OR nodularmelanom OR lentigomalignamelanom OR maligntmelanom* OR "#maligntmelanom" OR Akrallentiginousmelanom OR nevoidmelanom OR spitzoidmelanom OR desmoplastisktmelanom OR kutantmelanom) |

***** Calibrated by a team of Novartis Medical and external experts.
