# Supplementary material for: Understanding the Lived Experiences of Patients With Melanoma: Real-World Evidence Generated Through a European Social Media Listening Analysis
Source: JMIR Cancer. 2022 Jun 13;8(2):e35930. doi: 10.2196/35930 (PMC9237767; doi:10.2196/35930)
Supplement: Multimedia Appendix 2 [file cancer_v8i2e35930_app2.docx]

*Multimedia Appendix 2. Sources of Forum and Blog Posts.*

| **Type of Social Network** | **URL** |
| --- | --- |
| Forum | [https://www.mumsnet.com](https://www.mumsnet.com/) |
| Forum | <https://forum.frauenselbsthilfe.de/forum> |
| Forum | [https://www.krebs-kompass.de](https://www.krebs-kompass.de/) |
| Forum | <https://www.onmeda.de/forum> |
| Forum | <https://www.med1.de/forum> |
| Forum | [https://www.gutefrage.net](https://www.gutefrage.net/) |
| Forum | [https://forum.hardware.fr](https://forum.hardware.fr/) |
| Forum | <https://www.carenity.com/forum> |
| Forum | [https://forum.doctissimo.fr](https://forum.doctissimo.fr/) |
| Forum | [http://forum.dvdpascher.net](http://forum.dvdpascher.net/) |
| Forum | [https://forum.fiv.fr](https://forum.fiv.fr/) |
| Forum | [http://forum-hifi.fr](http://forum-hifi.fr/) |
| Forum | [https://communaute.ricaud.com](https://communaute.ricaud.com/) |
| Forum | <https://planete-homeopathie.org/forum> |
| Forum | <http://www.referendar.de/forum> |
| Forum | [http://beagleforum.de](http://beagleforum.de/) |
| Forum | [https://forum.prostatakrebs-bps.de](https://forum.prostatakrebs-bps.de/) |
| Forum | <http://www.stock-world.de/forum> |
| Forum | [https://community.myfitnesspal.de](https://community.myfitnesspal.de/) |
| Forum | [https://www.team-andro.com](https://www.team-andro.com/) |
| Forum | [https://www.gesundheitsfrage.net](https://www.gesundheitsfrage.net/) |
| Forum | [https://dcig-forum.de](https://dcig-forum.de/) |
| Forum | [https://www.babyforum.at](https://www.babyforum.at/) |
| Forum | [http://www.krebs-kompass.org](http://www.krebs-kompass.org/) |
| Forum | <https://www.bbhomepage.com/forum> |
| Forum | [http://www.durecomemuri.it](http://www.durecomemuri.it/) |
| Forum | <http://www.panperfocaccia.eu/forum> |
| Forum | <http://www.sosfegato.it/forumhcv> |
| Forum | [http://www.elsitodesandro.it](http://www.elsitodesandro.it/) |
| Forum | <https://www.9lives.be/forum> |
| Forum | <https://www.ouders.nl/forum> |
| Forum | [https://www.wereldfietser.nl](https://www.wereldfietser.nl/) |
| Forum | <https://www.familjeliv.se/forum> |
| Forum | [https://www.titleist.se](https://www.titleist.se/) |
| Forum | [http://forum.boundanna.net](http://forum.boundanna.net/) |
| Forum | <http://foorumi.h-y.fi/> |
| Forum | [http://foros.acb.com](http://foros.acb.com/) |
| Forum | [https://www.forocoches.com](https://www.forocoches.com/) |
| Forum | [https://www.burbuja.info](https://www.burbuja.info/) |
| Blog | [https://entertainment-today.be](https://entertainment-today.be/) |
