# Supplementary material for: Understanding the Lived Experiences of Patients With Melanoma: Real-World Evidence Generated Through a European Social Media Listening Analysis
Source: JMIR Cancer. 2022 Jun 13;8(2):e35930. doi: 10.2196/35930 (PMC9237767; doi:10.2196/35930)
Supplement: Multimedia Appendix 3 [file cancer_v8i2e35930_app3.docx]

*Multimedia Appendix 3. Pre-defined Inclusion/Exclusion Criteria.*

| **Inclusion criteria** | Posts **INCLUDED** in the final analysis:   - Contained information about melanoma, specifically; causes, symptoms experienced, diagnosis, treatment, management, disease endpoint (remission, recurrence, death), QoL impact, and unmet needs |
| --- | --- |
| **Exclusion criteria** | Posts **EXCLUDED** in the final analysis:   - Contained buy/sell content or market reports - Contained animal content - Were job postings - Were link duplicates - Only contained non-insightful content; simple mentions of melanoma without meaningful insight |
