# Supplementary figures and images for: Understanding the Lived Experiences of Patients With Melanoma: Real-World Evidence Generated Through a European Social Media Listening Analysis
Source: JMIR Cancer. 2022 Jun 13;8(2):e35930. doi: 10.2196/35930 (PMC9237767; doi:10.2196/35930)

*Multimedia Appendix 4.* *Analysis Process for Post Relevancy.*

**
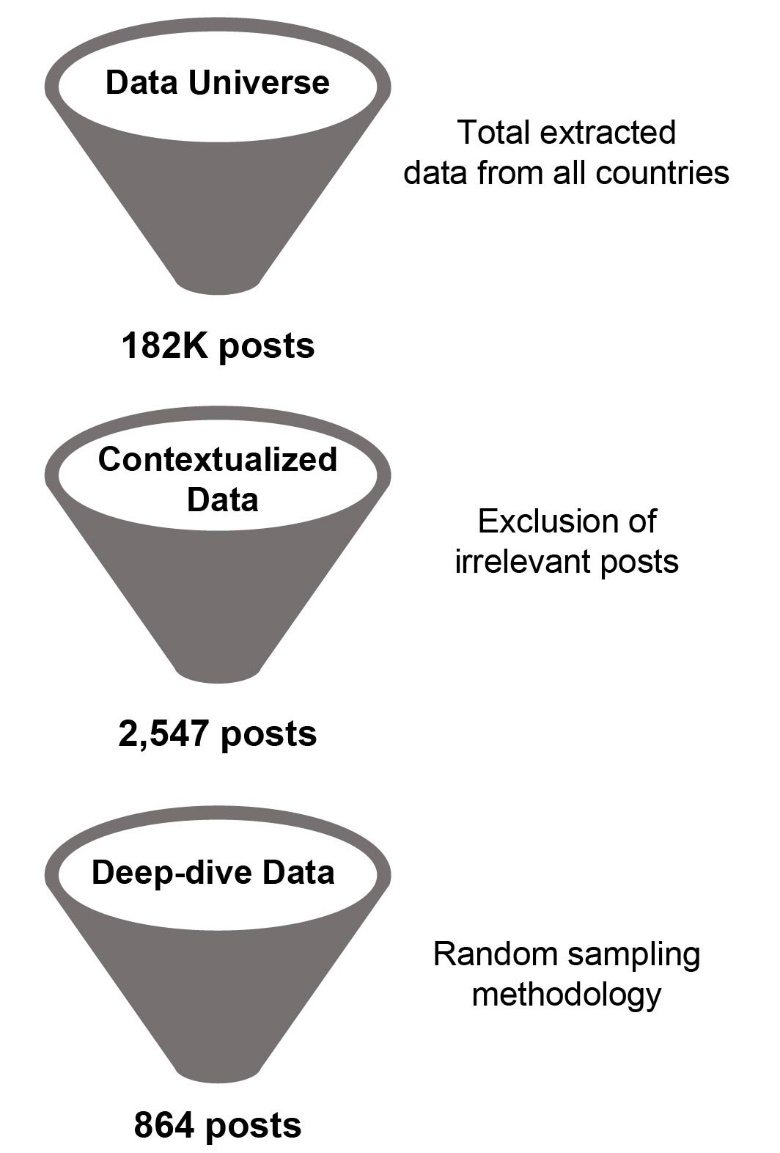
**

Supplement: Multimedia Appendix 4 [file cancer_v8i2e35930_app4.docx]

*Multimedia Appendix 5.* *Stakeholder Demographics by Country.*

**
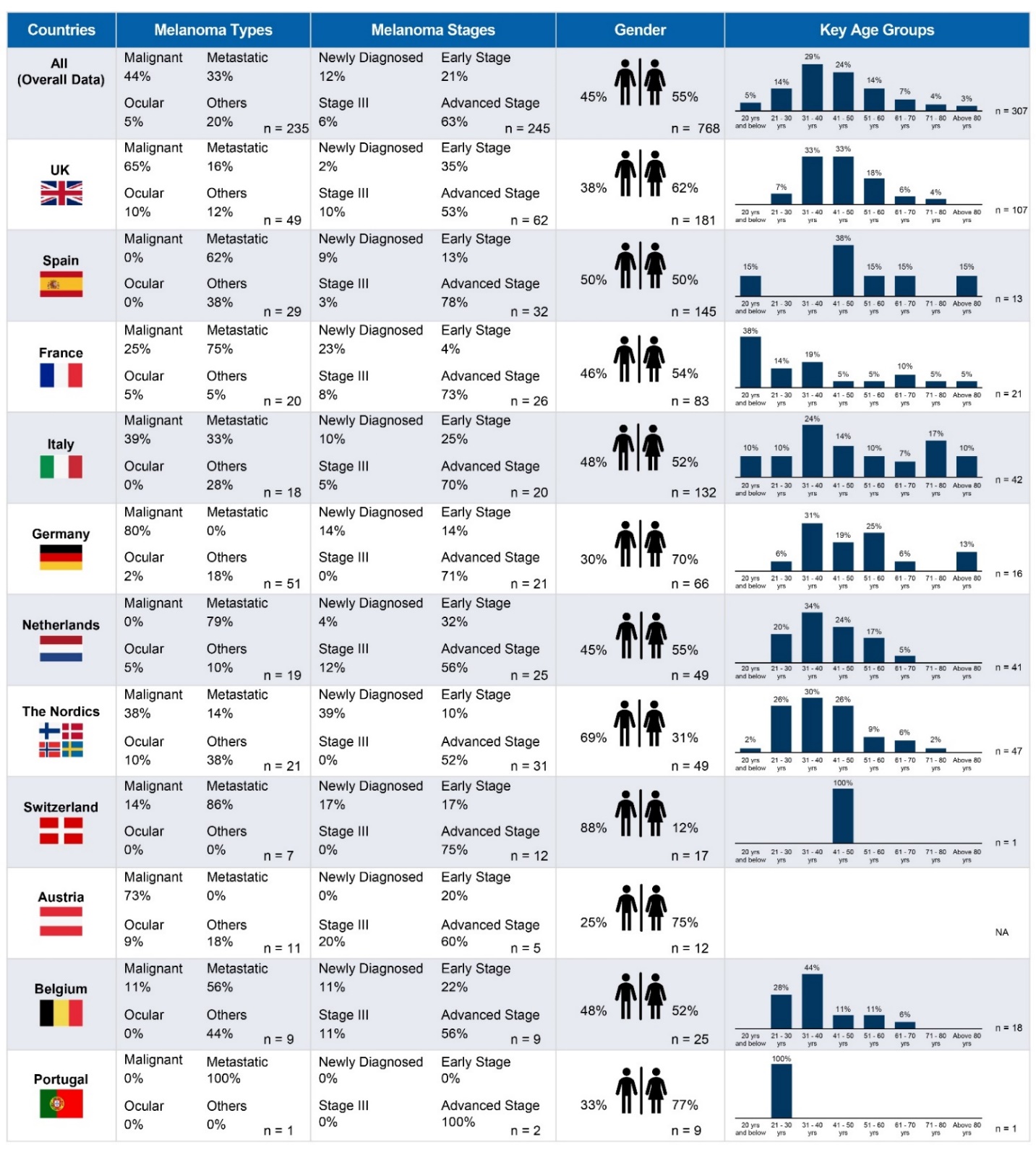
**

Supplement: Multimedia Appendix 5 [file cancer_v8i2e35930_app5.docx]

*Multimedia Appendix 8.* *Melanoma Treatment Discussions.*


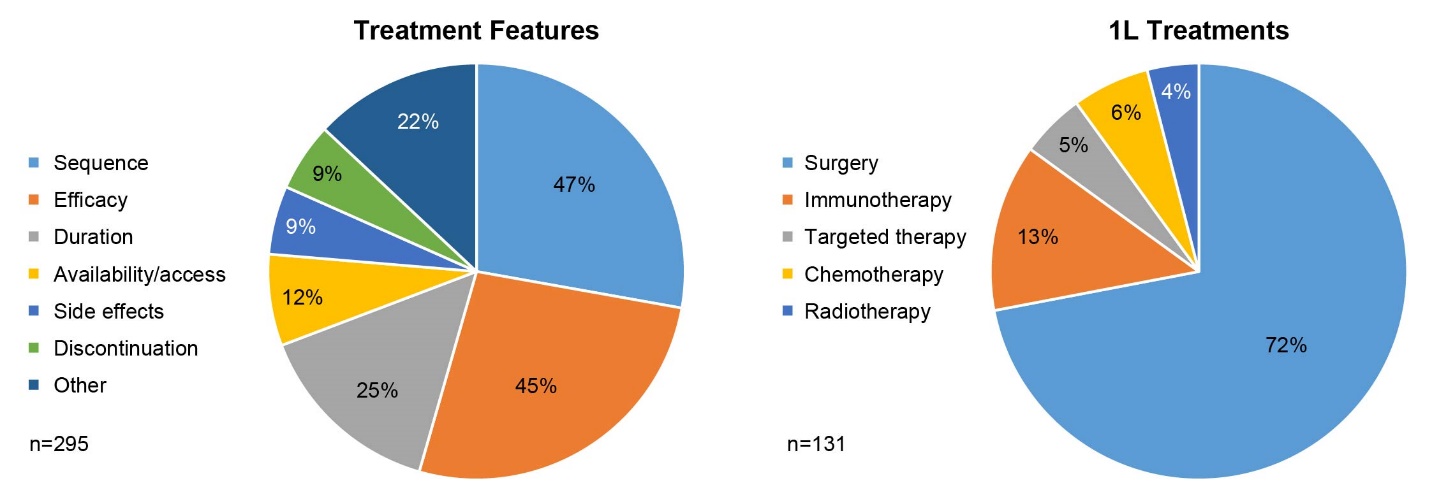

Supplement: Multimedia Appendix 8 [file cancer_v8i2e35930_app8.docx]

*Multimedia Appendix 9.* *Key Unmet Needs of Melanoma Stakeholders.*

**
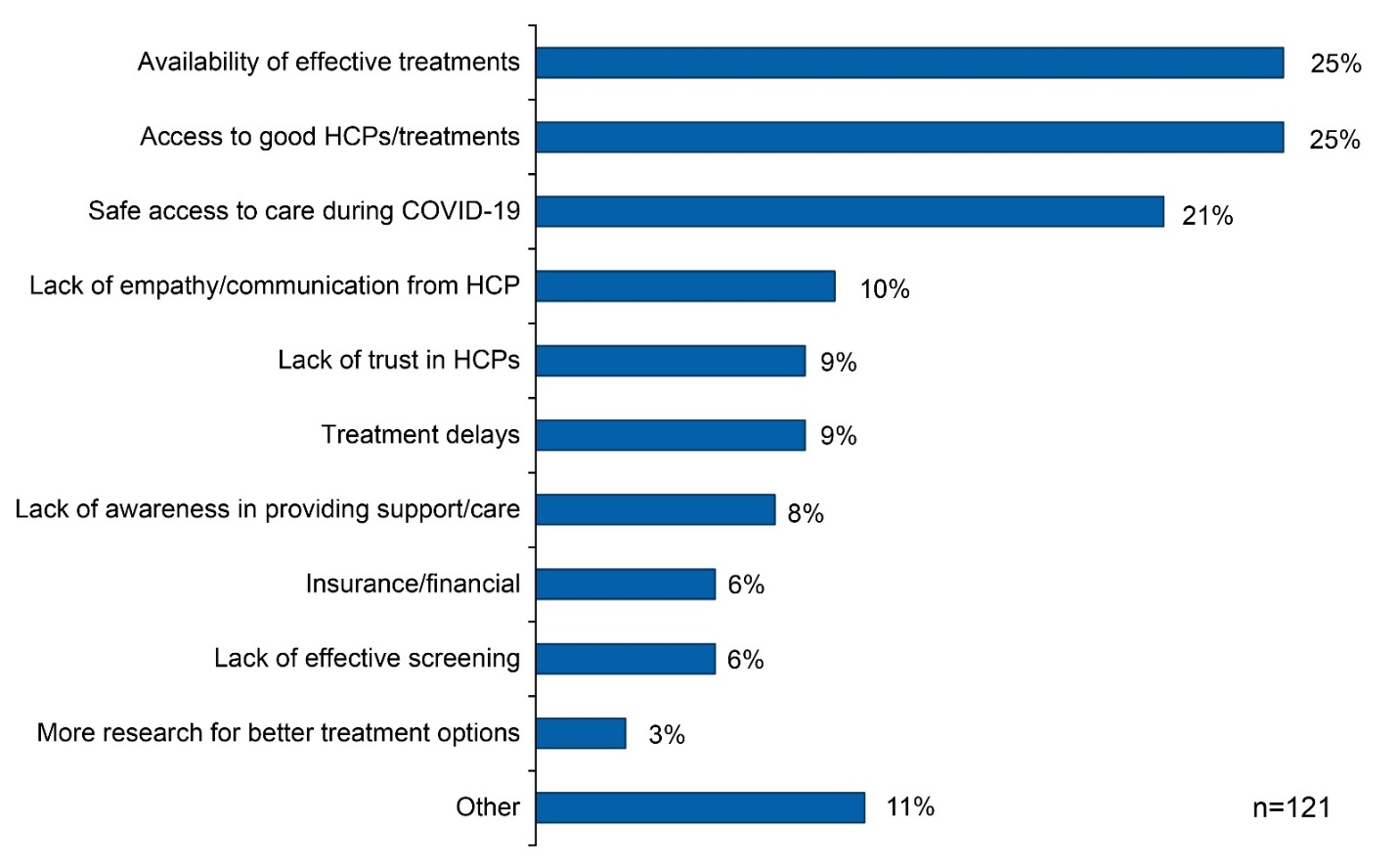
**

Supplement: Multimedia Appendix 9 [file cancer_v8i2e35930_app9.docx]
