# Supplementary material for: Understanding the Lived Experiences of Patients With Melanoma: Real-World Evidence Generated Through a European Social Media Listening Analysis
Source: JMIR Cancer. 2022 Jun 13;8(2):e35930. doi: 10.2196/35930 (PMC9237767; doi:10.2196/35930)
Supplement: Multimedia Appendix 6 [file cancer_v8i2e35930_app6.docx]

*Multimedia Appendix 6.* *Percentage of Melanoma Stakeholder Discussion on Diagnosis and Tests.*

**Percentage of Melanoma Stakeholder Discussion on Diagnosis and Tests.**

|  | n | Confirmed  Diagnosis (%) | Screening and Diagnostic Tests in General (%) | Diagnosis in General (%) | Biopsy (%) | Others (%) |
| --- | --- | --- | --- | --- | --- | --- |
| **Countries** (All) | **2555** | **36** | **20** | **15** | **18** | **40** |
| UK | 23 | 22 | 22 | 35 | 26 | 4 |
| Spain | 44 | 73 | 5 | 2 | 2 | 20 |
| France | 8 | 0 | 25 | 25 | 13 | 50 |
| Italy | 33 | 0 | 61 | 12 | 12 | 33 |
| Germany | 70 | 40 | 9 | 4 | 23 | 66 |
| Netherlands | 26 | 42 | 27 | 12 | 23 | 58 |
| Nordic countries | 20 | 20 | 0 | 55 | 35 | 25 |
| Switzerland | 5 | 0 | 20 | 40 | 20 | 40 |
| Austria | 15 | 60 | 27 | 0 | 0 | 40 |
| Belgium | 10 | 20 | 40 | 30 | 40 | 20 |
| Portugal | 1 | 0 | 0 | 100 | 0 | 0 |
