# Supplementary material for: Understanding the Lived Experiences of Patients With Melanoma: Real-World Evidence Generated Through a European Social Media Listening Analysis
Source: JMIR Cancer. 2022 Jun 13;8(2):e35930. doi: 10.2196/35930 (PMC9237767; doi:10.2196/35930)

*Multimedia Appendix 7. Melanoma Treatments Reported in Social Media Posts and Associated Sentiment. A. Type of melanoma treatments reported. B. Melanoma treatments as discussed by sentiment.*


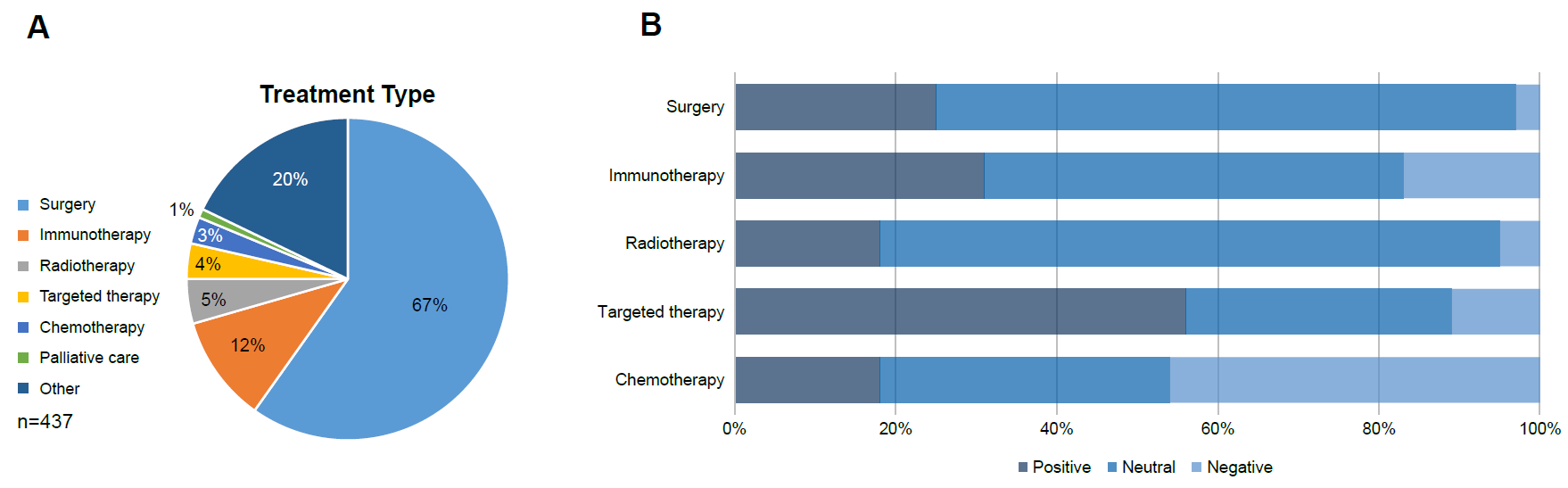

Supplement: Multimedia Appendix 7 [file cancer_v8i2e35930_app7.docx]
